# *In Vitro* Anthelmintic Activity of *Baliospermum montanum* Muell. Arg roots

**DOI:** 10.4103/0250-474X.40352

**Published:** 2008

**Authors:** R. G. Mali, R. R. Wadekar

**Affiliations:** Department of Pharmacognosy and Phytochemistry, Smt. S.S. Patil College of Pharmacy, Chopda (Jalgaon) - 425 107, India

**Keywords:** *Baliospermum montanum*, anthelmintic activity, *Pheretima posthuma*, *Ascardia galli*

## Abstract

Alcohol and aqueous extracts from the roots of *Baliospermum montanum* Muell. Arg were investigated for their anthelmintic activity against *Pheretima posthuma* and *Ascardia galli*. Various concentrations (10-100 mg/ml) of each extract were tested in the bioassay, which involved determination of time of paralysis and time of death of the worms. Both the extracts exhibited significant anthelmintic activity at highest concentration of 100 mg/ml. Piperazine citrate (10 mg/ml) was included as standard reference and distilled water as control.

*Baliospermum montanum* Muell. Arg (Family: Euphorbiaceae) commonly known as *Danti*, is a leafy, monoecious under shrub distributed throughout India, Burma and Malaya^1^. All parts of the plant like leaves, seeds and roots have been traditionally used to relieve variety of ailments. Decoction of leaves is reported to be useful in asthma and expressed juice of young leaves is applied to a bleeding cut while leaves are applied as a bandage which stops haemorrhage, prevents suppuration and heals the wound. The seeds are used as a drastic purgative and seed oil as powerful hydragogue cathartic and applied externally in rheumatism. In Ayurveda, roots of the plant are reported to be useful in jaundice, and in traditional system of medicine highly valued for treatment of leucoderma, piles, wound, anaemia, itching, pains and inflammations and reputed as an anthelmintic^2^–^4^. Earlier reports on pharmacological activity of the roots are scarce. In the present study, anthelmintic potential of alcoholic and aqueous extracts of roots of *B. montanum* have been evaluated.

The roots of *B. montanum* were collected from Chopda, Maharashtra during October/November 2005. The roots were identified and authenticated by the Department of Botany, SSVPS's LK Dr. P.R. Ghogrey Science College, Dhule, Maharashtra and a voucher specimen was deposited at the Department of Pharmacognosy, Smt. S. S. Patil College of Pharmacy, Chopda.

The roots were cleaned, shade dried and coarsely powdered. The coarse powder of roots was then exhaustively extracted in a Soxhlet apparatus. Ethyl alcohol was used as a solvent for alcoholic extract whereas distilled water for aqueous extract. The solvent was allowed to evaporate in a rotary vacuum evaporator. The dry extracts obtained were subjected to various chemical tests to detect the presence of different phytoconstituents^5^,^6^

*Pheretima posthuma* (Annelida), commonly known as earthworm were collected from the water logged areas and *Ascardia galli* (nematode) worms were obtained from freshly slaughtered fowls (*Gallus gallus*). Both worm types were identified at the P. G. Department of Zoology, Pratap College, Amalner.

The anthelmintic assay was carried as per the method of Ajaiyeoba *et al.*^7^ with minor modifications. The assay was performed on adult Indian earthworm, *Pheretima posthuma* due to its anatomical and physiological resemblance with the intestinal roundworm parasite of human beings^8^–^11^. Because of easy availability, earthworms have been used widely for the initial evaluation of anthelmintic compounds *in vitro*^12^–^16^. *Ascardia galli* worms are easily available in plenty from freshly slaughtered fowls and their use, as a suitable model for screening of anthelmintic drug was advocated earlier^17^–^19^. Fifty millilitre of formulation containing three different concentrations, each of crude alcoholic and aqueous extract (10, 50 and 100 mg/ml in distilled water) were prepared and six worms (same type) were placed in it. This was done for both types of worm. Time for paralysis was noted when no movement of any sort could be observed except when the worms were shaken vigorously. Time for death of worms was recorded after ascertaining that worms neither moved when shaken vigorously nor when dipped in warm water (50°). Piperazine citrate (10 mg/ml) was used as reference standard while distilled water as control^20^–^21^.

Preliminary phytochemical screening of alcoholic extract revealed the presence of alkaloids, tannins, phenolic compounds and steroids while aqueous extract showed presence of phenolic compounds and tannins. As shown in Table 1, the alcoholic and aqueous extracts of roots of *B. montanum* displayed significant anthelmintic properties at higher concentrations. Both the extracts showed anthelmintic activities in dose-dependant manner giving shortest time of paralysis (P) and death (D) with 100 mg/ml concentration, for both type of worms. The alcoholic extract of *B. montanum* caused paralysis in 10 min and death in 28 min, while aqueous extract showed P and D in 9 and 30 min. against the earthworm *P. posthuma*. The reference drug piperazine citrate showed the same at 21 min and 59 min.

**TABLE 1:** ANTHELMINTIC ACTIVITY OF ALCOHOL AND AQUEOUS EXTRACT OF *BALIOSPERMUM MONTANUM*

Test subs	Concentration (mg/ml)	Time taken for paralysis (P) and death (D) of worms in min			
		
		*P. posthuma*		*A. galli*	
			
		P	D	P	D
Control	-	-	-	-	-
Alcohol extract	10	23 ± 0.1	63 ± 0.4	16 ± 0.6	45 ± 0.1
	50	16 ± 0.4	43 ± 0.6	08 ± 0.8	33 ± 0.5
	100	10 ± 0.2	28 ± 0.8	28 ± 0.8	29 ± 0.6
Aqueous extract	10	25 ± 0.1	66 ± 0.3	17 ± 0.2	48 ± 0.6
	50	18 ± 0.7	48 ± 0.2	10 ± 0.6	36 ± 0.9
	100	09 ± 0.8	30 ± 0.1	06 ± 0.6	27 ± 0.2
Piperazine citrate	10	21 ± 0.2	59 ± 0.6	12 ± 0.01	41 ± 0.4

Results are expressed as mean ± SEM from six observations

*Ascardia galli* worms also showed sensitivity to the alcoholic and aqueous extracts of *B. montanum*. The alcoholic extract caused paralysis in 5 min, death in 29 min and the aqueous extract displayed P and D in 6 and 27 min, respectively, at higher concentration of 100 mg/ml. Piperazine citrate did the same at 12 and 41 min.

The predominant effect of piperazine citrate on the worm is to cause a flaccid paralysis that result in expulsion of the worm by peristalsis. Piperazine citrate by increasing chloride ion conductance of worm muscle membrane produces hyperpolarisation and reduced excitability that leads to muscle relaxation and flaccid paralysis^22^. The root extract of *B. montanum* not only demonstrated paralysis, but also caused death of worms especially at higher concentration of 100 mg/ml, in shorter time as compared to reference drug piperazine citrate. Phytochemical analysis of the crude extracts revealed presence of tannins as one of the chemical constituent. Tannins were shown to produce anthelmintic activities^23^. Chemically tannins are polyphenolic compounds^24^. Some synthetic phenolic anthelmintics e.g. niclosamide, oxyclozanide and bithionol are shown to interfere with energy generation in helminth parasites by uncoupling oxidative phosphorylation^25^. It is possible that tannins contained in the extracts of *B. montanum* produced similar effects. Another possible anthelmintic effect of tannins is that they can bind to free proteins in the gastrointestinal tract of host animal^26^ or glycoprotein on the cuticle of the parasite^27^ and cause death.

In conclusion, the traditional claim of roots of *Baliospermum montanum* as an anthelmintic have been confirmed as the root extracts displayed activity against the worms used in the study. Further studies to isolate and reveal the active compound (S) contained in the crude extracts of *B. montanum* and to establish the mechanism (S) of action are required.
